# Does “National Civilized City” policy mitigate air pollution in China? A spatial Durbin difference-in-differences analysis

**DOI:** 10.1186/s12889-024-18671-y

**Published:** 2024-05-04

**Authors:** Lei Jiang, Zinan Zhang, Bo Zhang, Shixiong He

**Affiliations:** 1 Center for Human Geography and Urban Development, School of Geography and Remote Sensing, Guangzhou University, Guangzhou, 510006 China; 2 Guangdong Provincial Center for Urban and Migration Studies, Guangzhou, 510006 China; 3 School of Public Finance and Taxation, Zhejiang University of Finance and Economics, Hangzhou, 310018 China; 4 Key Research Center of Philosophy and Social Sciences of Zhejiang Province, The Institute of Local Finance Research, Zhejiang University of Finance and Economics, Hangzhou, 310018 China; 5 School of Urban and Regional Science, Institute of Finance and Economics Research, Shanghai University of Finance and Economics, Shanghai, 200433 China

**Keywords:** National Civilized City, SO_2_ pollution, Spatial spillovers, Satellite observed data, Spatial Durbin difference-in-differences model, Robustness check

## Abstract

“National Civilized City” (NCC) is regarded as China’s highest honorary title and most valuable city brand. To win and maintain the “golden city” title, municipal governments must pay close attention to various key appraisal indicators, mainly environmental ones. In this study we verify whether cities with the title are more likely to mitigate SO_2_ pollution. We adopt the spatial Durbin difference-in-differences (DID) model and use panel data of 283 Chinese cities from 2003 to 2018 to analyze the local (direct) and spillover effects (indirect) of the NCC policy on SO_2_ pollution. We find that SO_2_ pollution in Chinese cities is not randomly distributed in geography, suggesting the existence of spatial spillovers and possible biased estimates. Our study treats the NCC policy as a quasi-experiment and incorporates spatial spillovers of NCC policy into a classical DID model to verify this assumption. Our findings show: (1) The spatial distribution of SO_2_ pollution represents strong spatial spillovers, with the most highly polluted regions mainly situated in the North China Plain. (2) The Moran’s I test results confirms significant spatial autocorrelation. (3) Results of the spatial Durbin DID models reveal that the civilized cities have indeed significantly mitigated SO_2_ pollution, indicating that cities with the honorary title are acutely aware of the environment in their bid to maintain the golden city brand. As importantly, we notice that the spatial DID term is also significant and negative, implying that neighboring civilized cities have also mitigated their own SO_2_ pollution. Due to demonstration and competition effects, neighboring cities that won the title ostensibly motivates local officials to adopt stringent policies and measures for lowering SO_2_ pollution and protecting the environment in competition for the golden title. The spatial autoregressive coefficient was significant and positive, indicating that SO_2_ pollution of local cities has been deeply affected by neighbors. A series of robustness check tests also confirms our conclusions. Policy recommendations based on the findings for protecting the environment and promoting sustainable development are proposed.

## Introduction

China’s rapid economic growth, which is characterized by extensive development, has depended largely on natural resources, particularly fossil fuels [1]. In 2009, China became the largest energy consumer in the world [2], with coal being the dominant energy source for over 40 years. In 2021, in terms of coal consumption, China accounted for more than 50% of the world total [3]. A main pollutant of coal consumption is sulfur dioxide (SO_2_), which has led to widespread and serious environmental pollution problems [4], SO_2_ is a toxic gas that not only poses a threat to people’s health but also damages sustainable development [5]. In other words, China’s economic growth has been accompanied by serious environmental degradation at the expense of people’s health and the environment. In response, both the central and local governments have taken steps to strengthen various policies and measures to mitigate pollution, notably SO_2_ pollution [6]. For example, in the 11th Five-year-plan period (2006–2010), central government set up a 5-year short-term binding and quantitative target for SO_2_ emissions reduction. Specifically, national SO_2_ emissions were enforced to reduce by 10% [7]. The target was reinforced in the 12th FYP with a further 8% reduction than that of 2010. The two targets were successfully achieved by reducing 14.29% and 18% by the end of the 11th and 12th FYPs [8], signaling strong environmental awareness and dedication by governments to tackle the environmental degradation, reduce SO_2_ pollution, and realize the goal of sustainable development in Chinese cities.

To promote harmonious and sustainable development and enhance environmental governance, the Central Commission for Guiding Cultural and Ethical Progress published the “*National Civilized City Appraisal Systems (Trial)*,” to motivate local government participation in a competition in a non-economic field, e.g., environmental protection, with the aim to achieve greater public service [9]. It implies a transition of the emphasis of government on public services rather than only economic performance, e.g., GDP tournaments [10]. Environmental performance has become one of the critical indicators, e.g., an environmental policy tool, of central government to promote an ecological civilization. Notably, one of its goals is to reduce pollution and accelerate sustainable development. Since it is a high-profile program run by the Central Commission, the NCC is regarded to date as the highest honor among Chinese cities and the most valuable city brand in China [1], it is also the most difficult award to receive [9]. Cities winning the award are usually referred to as *civilized cities*. Municipal officials of all Chinese cities therefore pay close attention to this phenomenon. On the one hand, it will greatly enhance political performance of the municipal officials and is beneficial to prestige and promotion in economically developed cities that have already won the golden title. Liu [11] reports that more than a half of mayors of civilized cities have received promotions, which are generally referred to as a promotion tournament [12].

On the other hand, since the weight of economic indicators accounts for less than 3%, much lower than that of environmental management and quality (7.89%), it provides a novel competition model that signals a clear shift from “competition for rapid growth” to “competition for harmonious development.” In this sense, those officials of less economically developed cities but with high level of environmental protection and sustainability, also have opportunities to demonstrate their governance practices and performance. Hence, the NCC policy can also mobilize the initiatives of local governments to improve cities. In fact, this place-based competition is prevalent in China. More specifically, it is an institutionalized competition contest model, where the top central commission establishes measurable, easy-to-evaluate, and easy-to-compare criteria and sets clear-cut goals to make lower local governments compete. Afterwards, the central commission assesses and awards the winners [13] in what is usually referred to as the top-down place-competition and award model [14]. This practice still maintains a sufficient incentive effect on local officials and has become a larger contributor to tenure experience and political promotion than that of economic performance. In 2005, the first batch of cities with the NCC title was evaluated and recognized by the Central Commission [15]. Since then, a total of 146 cities (including 25 provincial capital cities and sub-provincial cities, and 121 prefecture-level cities) have been selected in the NCC campaign and the competition for this honorary title has intensified year on year.

The appraisal system for the NCC has been well-developed after several revisions over the past 20 years. At present it consists of 3 modules, 12 indicators and 90 sub-indicators [12], and emphasizes public service sectors, like the ecological environment. According to the “National Civilized City Appraisal System (2007 Edition),” the basic indicators in public service concentrate on two major aspects, namely, the environment for citizens’ work and life, and the ecological civilization for sustainable development. In the 11th version of 2015, the environment-related indicators in the appraisal system expanded to include nine green and low-carbon sub-indicators, e.g., urban air quality [16]. In other words, sustainable development is the most important among the evaluation indicators [17] because the problem of environmental degradation has become increasingly prominent in recent decades, which has had a remarkable influence on the selection of the NCC. Also, the Central Commission inspects the progress of environmental improvements in field visits, making local governments focus on livelihood and environmental indicators. Most importantly, the golden title cannot be permanently held by the civilized cities, after receiving the award, they are reviewed in each subsequent assessment. The title is revoked if indicators such as environmental concerns fail to meet the standards. Given that the successful rating of civilized city could significantly increase the likelihood of promotion, during the tournament, local governments must strenuously improve all the indicators, e.g., environmental quality, to maintain the honorary city brand. Otherwise, local governments of civilized cities are likely to have their golden title withdrawn. The implication here is that the competition carries on long-term and will continue until there is no room for improvement. Also, the NCC appraisal has the unique characteristic of not always being a one-time process. The cities participating in the competition must undergo several selection processes before finally winning the honorary title [18], this can fully motivate local officials to stay competitive. In this sense, it is safe to say that this institutional design is robust because it addresses and copes with problems such as environmental pollution which cannot be solved in the short-term.

We inquire whether cities winning the NCC title may significantly mitigate SO_2_ pollution of their own city and also their neighbors, since the golden title is an incentive to resolve environmental pollution and improve the environmental quality for the purpose of political promotion of municipal officials. However, the performance contest does not work across the full range of China’s political systems; it is applicable only to certain institutional arenas. Hence, it is necessary to assess the effect of the NCC policy on the environment. Whereas the NCC contest can also be regarded as another promotion tournament, in which place-based officials win the honorary title through mutual competition for promotion opportunities. In this way, a civilized city may stimulate its neighbors to participate in the tournament and improve assessment indicators, such as environmental problems, which are referred to as demonstration effects. To answer this question and to capture the demonstration effect, we treat the NCC as a quasi-experiment and introduce the spatial Durbin DID model to verify this hypothesis of the local and spillover effects of the NCC policy on mitigating SO_2_ pollution.

The research is organized such that "Literature review" section gives a literature review and "Methods and data sources" section introduces methods and data sources. "Empirical results and discussion" section presents empirical results and robustness checks while "Discussion" section summarizes our conclusions and proposes policy implications.

## Literature review

Evidence indicates that the mutual competition to gain the civilized city title, as a public service motivation tool, is advantageous in coping with public issues [14]. The NCC as a policy tool is therefore increasingly being applied in the literature, especially towards quantifying its impacts. Studies can be divided into three strands. In the first strand some researchers focus on the impact of the NCC in the competition of economic performance. For example, Huang and Zhou [19] chose two civilized cities, both having received five consecutive honorary titles: Baotou and Yantai, and adopted a synthetic control method. Results show that the NCC has had a long-term and positive impact on economic growth. In another study, Chen and Mao [20] used a quasi-experiment approach to evaluate the NCC on tourism. The findings indicate that the NCC title indeed contributes to growth in the tourism economy of civilized cities. The Li et al. [12] study showed that civilized cities exhibit significant improvements in public services as well as the subjective perceptions of citizens. The NCC award was also shown to have a positive effect on the promotion of local leaders. Wang and Wang [21] found that civilized cities substantially increase the scale of municipal investment debt. Further studies found evidence that taking the NCC into consideration also contributes to increasing house prices [22], attracting migrants [23], industrial upgrading [24], urban economic growth [25], and urban green innovation [9], among others.

The second strand focuses on evaluating the effect of the NCC award on local enterprise performance. For example, Wu et al. [26] found that having NCC status lowers transaction costs, thereby increasing the profitability of local privately listed firms. Shi et al. [27] concluded that the NCC promotes the total factor productivity and labor productivity of local firms. Qi et al. [28] confirmed that the NCC policy increases the corporate environmental, the social governance performance of civilized cities. Zhao et al. [29] applied a staggered DID model and investigated whether the NCC decreased the risk of stock price crash for local firms. Their results indicated that it could significantly reduce future crash risk. Apart from the objective indicators of firms, the NCC may also have an impact on subjective perceptions since it emphasizes the construction of a spiritual civilization. For example, a study by Chai et al. [30] revealed that the honorary title enhances the sense of corporate social responsibility.

The third strand identifies the effect of the NCC in non-economic areas, such as environmental performance and green development. The mechanism of the NCC competition is beneficial for solving the environmental degradation problem caused by the traditional GDP tournament [13]. For example, Lu et al. [31] adopted a propensity score matching and difference-in-differences (PSM-DID) approach to examine the impact of NCC on environmental pollution and find that it could contribute to improving urban environments. Zhang et al. [15] also applied the PSM-DID approach based on a quasi-experiment of participating in the NCC campaign,they evaluated the effect of government intervention on corporate environmental performance. Their results show that the environmental performance of firms in civilized cities is higher than in non-civilized ones. Similarly, Shi et al. [32] also adopted the PSM-DID approaches, using panel data of 281 cities from 2000 to 2018 and find that the NCC designation of the cities is likely to greatly improve the green total factor productivity. Li et al. [12] highlighted the impact of NCC on the energy efficiency of resource-based cities in China and found that NCC is beneficial to energy efficiency improvements. Lastly, Yang et al. [17] emphasized the effect of NCC on green innovation in Chinese cities. Green innovation is deemed capable of mitigating environmental pollution by increasing production efficiency to achieve a double dividend effect between urban economic growth and environmental sustainability. Yang et al. [17] found that NCC could contribute to increasing the level of urban green innovation.

From the literature we find that the NCC helps make cities better by enhancing the performance of local firms and improving the urban environment. However, very few studies pay direct attention to the impact of the NCC on pollutant reduction. One exception is a study by Shen et al. [33] who use panel data of manufacturing firms from 1999 to 2008 in China to calculate the environmental effect of the NCC at firm level from a micro perspective. Their results showed that firms in civilized cities have reduced more COD discharge than firms in non-civilized cities. In another study, Huang et al. [16] have evaluated the reduction effect of the NCC on carbon emissions, and found that it significantly lowered emissions with the reduction effect strengthening over time. However, we observe that spatial spillovers have not yet been considered in empirical studies when examining the impact of the NCC on pollution reduction. The two studies also suffer from two shortcomings. One is that spatial spillovers of pollutants was omitted in the Shen et al.’s [33] study. Since pollution in Chinese cities is not randomly distributed, the omission of spatial spillovers may also lead to biased conclusions. The second is that statistical data may not objectively and accurately measure the pollution level of Chinese cities, since pollutant data, one of the key performance indictors to determine the promotion of local officials, could be modified or falsified during data collection [8]. Recently, satellite observed pollutant data, such as SO_2_ concentrations, have been widely studied thanks to two main advantages, namely, the ability to objectively describe SO_2_ pollution, and full coverage. In this regard, we introduce satellite observed SO_2_ pollution to perform a robustness check.

Correspondingly, the contributions of this research may be threefold. Firstly, we incorporate spatial spillovers of both SO_2_ pollution and the NCC and then make use of the NCC campaign to build a spatial Durbin DID model to evaluate the direct and demonstration effects of the NCC on SO_2_ pollution reduction. Doing so provides evidence for understanding the environmental impact of government policies, e.g., the selection of civilized city, on non-economic performance indicators. In addition, the present research better clarifies policy boundaries and demonstration effects of the NCC policy and provides an underlying implication that the widening effect should be considered when designing and implementing an institutional policy. Secondly, in general, rook contiguity spatial weights matrix is the first to consider. When introducing spatial demonstration effects of the NCC in the model, it is a strong assumption that only cities sharing the common borders with civilized cities will be affected. In fact, it is likely that more neighboring cities may be affected due to demonstration effects, which also should be incorporated in the model. Hence, this research introduces different spatial weights matrices and constructs a multi-period spatial Durbin DID model to correct the possible multi-period bias from differential trends. Thirdly, we introduce satellite observed SO_2_ concentrations data to again confirm the environmental effects of the NCC on pollution reduction. We aim for our robust and convincing conclusions to contribute to eliminating underlying bias and formulating effective policies and measures. The main findings of this research also further clarify the mutual competition of the NCC campaign, thereby enriching the literature on the nexus between local governance and environmental performance. Lastly, the conclusions of this research may provide a novel insight into designing and implementing effective policies to improve environmental quality in China from the perspective of mutual competition.

## Methods and data sources

### Classical DID model

The NCC can be regarded an environmental policy tool. In this sense, we conduct a quasi-experiment and evaluate the impact of the NCC program on SO_2_ pollution. Specifically, our selected treatment group is cities that won the title of the NCC. Whereas the control group encompasses non-civilized cities. Hence, to ascertain the policy effect, we analyze if the control group (non-civilized cities) can be significantly distinguished from the treatment group (civilized cities) in terms of SO_2_ pollution reduction. To this end, we introduce the DID model because it can remove the bias between the control group and the treatment group [34].

Since the selection of NCC is a dynamic cycle process and program has been conducted for five times within our sample period, this provides us to use a staggered DID method. The key explanatory variable, *DID*_it_, is set to 1 if city *i* was a civilized city in year *t*, but otherwise, 0. The DID model can be written as

1 $$\begin{array}{c}{LnSO}_{2it}=a+\zeta {DID}_{it}+{\mu }_{i}+{\lambda }_{t}+{\varepsilon }_{it}\end{array}$$

where *Ln* denotes natural logarithm transformation. SO_2_ is the dependent variable, SO_2_ emissions. *DID* is the core variable. $\zeta$ is an unknown parameter to be estimated. If it is highly significant and negative, we can conclude that civilized city is able to significantly reduce SO_2_ pollution and captures the policy effect of the NCC. Furthermore, ${u}_{i}$ and ${\lambda }_{t}$ represent city fixed effects and time fixed effects, respectively. Lastly, $\varepsilon$ is an error term.

To avoid omitted variables biased conclusions, we also incorporate several control variables in the DID model, which can be re-written as

2 $$\begin{array}{c}{LnSO}_{2it}=a+\zeta {DID}_{it}+X\beta +{\mu }_{i}+{\lambda }_{t}+{\varepsilon }_{it}\end{array}$$

where *X* denotes a set of control variables, including gross urban product (*GUP*), GUP growth rate (*GUPr*), population (*POP*), and foreign direct investment (*FDI*). The other variables are the same as Eq. (1).

### Spatial DID model

The classical DID model assumes that observations are independent from each other. However, findings indicate that the observations of proximate spatial units tend to have similar values in which, technically speaking, there may be spatial spillovers. In other words, a city suffering SO_2_ pollution is deeply affected by neighbors. The potential spatial spillovers of the observations should therefore be considered in the model; not doing so could lead to biased conclusions.

In general, two spatial econometric models are commonly used in empirical studies, namely, spatial lag model and spatial error model. The former considers spatial spillovers of our dependent variable, namely, SO_2_ pollution which is expressed as

3 $${LnSO}_{2it}=a+\zeta {DID}_{it}+\rho {WLnSO}_{2it}+{X}_{it}\beta +{\mu }_{i}+{\lambda }_{t}+{\varepsilon }_{it}$$

where *WLnSO*_*2it*_ is the spatially lagged dependent variable used to capture spatial spillovers of SO_2_ pollution. $\rho ,$ also known as spatial autoregressive coefficient, is an unknown parameter to be estimated. *W* is a n × n spatial weights matrix used to describe the arrangement of spatial units (i.e., Chinese cities). In this study we adopt two types of commonly used distance-based matrices, e.g., inverse distance matrix and k-nearest matrix. The former refers to a matrix with element ${w}_{ij}=1/{d}_{ij}$, where *d* is the distance between pairs of Chinese cities. However, in practice its disadvantage is that large distances will yield small values of the matrix, and vice versa. To overcome this shortcoming, we set a cut-off criterion, *δ*, such that ${w}_{ij}=0$ for ${d}_{ij}>\delta$. We consider four cut-off values, namely, 300, 350, 400, and 450 km. The four inverse distance matrices are labeled inv300, inv350, inv400, and inv450, respectively. The latter is an alternative type of distance-based matrix. The difference is that k-nearest neighbor matrix considers that the closest cities can have a spatial spillover effect. k indicates the number of nearest neighbors. However, there is no pre-defined statistical method to help us determine the most favorable value of k. To obtain robust conclusions, we thus also consider four values, e.g., 5, 6, 7, and 8. Hence, the four k-nearest neighbor matrices are labeled k5, k6, k7, and k8, respectively.

The spatial error model highlights spatial dependence among disturbances by incorporating a spatially autocorrelated errors in the model. Similarly, the spatial spillovers of exogenous control variables should also be considered in the model because in econometrics, the exclusion of spatially lagged explanatory variables may also lead to omitted variables bias and even undermine the foundation of the research [35]. Hence, based on the spatial lag model, we can build another model (spatial Durbin) that includes spatially lagged explanatory variables. Another advantage of the spatial Durbin model is that it will not produce biased estimates even though data generation processes point to the spatial lag model or spatial error model [36]. Hence, it is more appropriate in empirical studies. Here, we adopt the spatial Durbin DID model to capture both spatial spillovers of SO_2_ pollution and exogenous control variables by adding the spatially lagged dependent and independent variables in the model. The spatial Durbin DID Model can be expressed as

5 $${LnSO}_{2it}=a+\zeta {DID}_{it}+\updelta {WDID}_{it}+\rho {LnSO}_{2it}+{X}_{it}\beta + {WX}_{it}\theta +{\mu }_{i}+{\lambda }_{t}+{\varepsilon }_{it}$$

where *WX* denotes the spatially lagged independent variables, and $\theta$ is unknown parameters to be estimated.

It is worth noting that, apart from *WX*, *WDID* in the spatial Durbin DID model captures the spatial spillover effects of the NCC. If the coefficient is statistically significant and negative, we can conclude that neighboring civilized cities also have impacts on the SO_2_ pollution reduction of the local city, indicating that the policy not only has a reduction effect on SO_2_ pollution of the local civilized city, but also exhibits a spatial spillover effect on SO_2_ pollution of neighboring cities.

### Data sources

The data for all variables is derived from China City Statistical Yearbooks. To obtain robust conclusions we also conduct a robustness check. Specifically, we replace the dependent variable, SO_2_ emissions, with satellite observed SO_2_ concentrations known as planetary boundary layer SO_2_ vertical column densities. These are retrieved from the Ozone Measurement Instrument onboard the EOS-Aura satellite [8, 37]. The description, units and descriptive statistics for the variables involved in this study, including mean, standard deviation (S.D.), Min (minimum), and Max (maximum) are summarized in Table 1.

**Table 1:** Descriptive statistics for variables involved in this study

Variable	Description	Unit	Mean	SD	Min	Max
*SO*_*2*_	Industrial SO_2_ emissions	Tons	53031.75	57553.78	2	683162
*DID*	Civilized city or not	Na	0.12	0.33	0	1
*Pop*	Population	10000	441.14	391.91	16.37	11098.4
*GDPgrate*	Growth rate of GUP	%	11.42	4.34	-19.38	37.69
*GUP*	Gross urban product	million Yuan	161.84	262.10	3.18	3267.99
*FDI*	Foreign direct investment	million USD	734.60	1964.01	0.02	30825.63
*FISC*	Ratio of fiscal revenue to expenditure	%	0.49	0.26	0.0464	8.3902

## Empirical results and discussion

### Spatial distribution of SO_2_ emissions

**Fig. 1:**
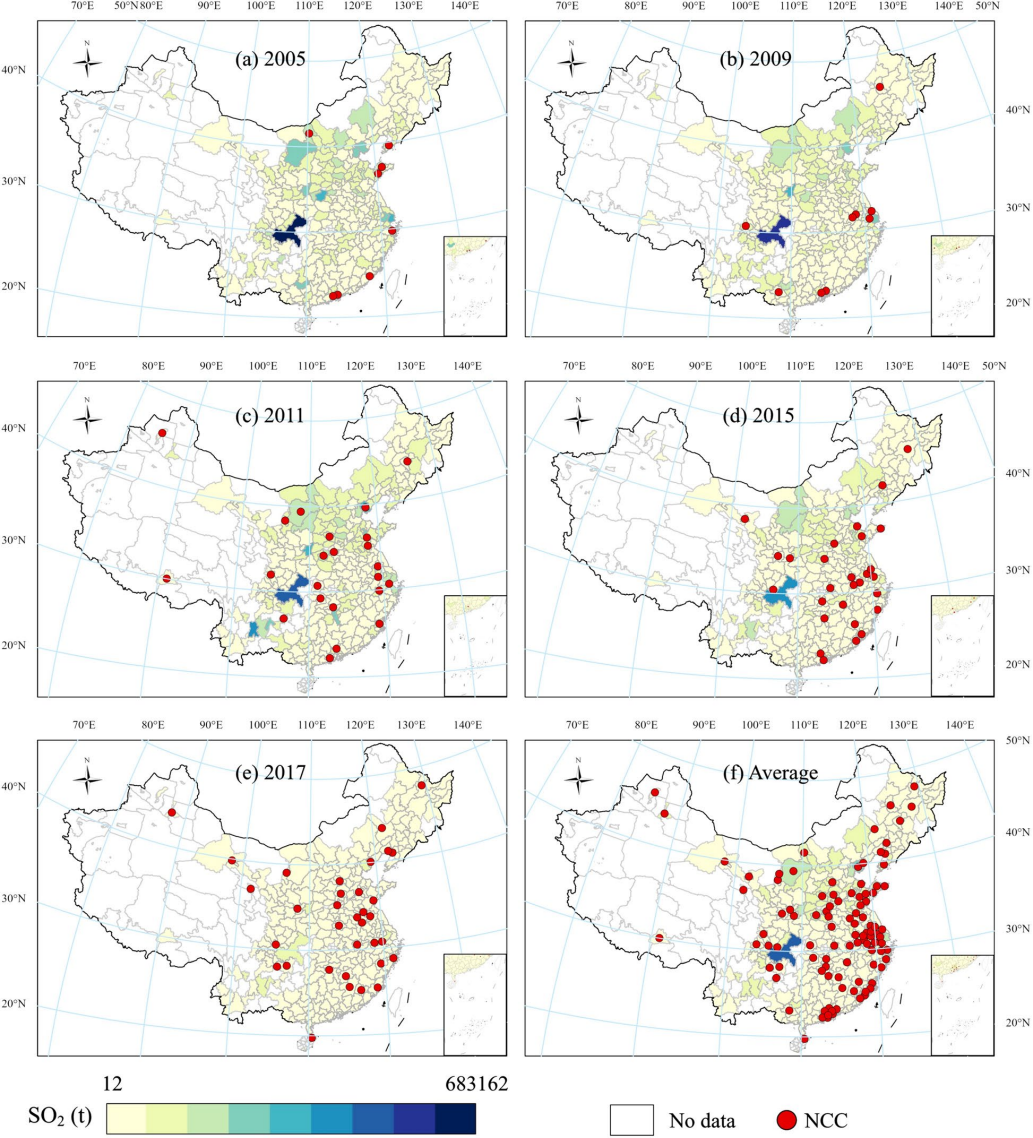
Spatial distribution of SO_2_ emissions and civilized cities in 2005, 2009, 2011, 2015, and 2017 as well as average emissions and total civilized cities

To better understand our application of the spatial Durbin DID model, the distribution of SO_2_ emissions of Chinese cities and the civilized cities are geo-visualized in Fig. 1 for years 2005, 2009, 2011, 2015, and 2017.

We can observe that in 2005 the SO_2_ emissions were not evenly distributed in China (Fig. 1a). The cities with the highest values mainly concentrated in the North China Plain, specifically, most of Hebei province, northern Henan province, and western Shandong province, as well as Baotou in Inner Mongolia; these highly polluted regions were characterized by heavy industries such as steel, which consumed enormous amounts of coal and emitted a million tons of SO_2_. Notably, Chongqing in the southwest had the highest emissions due to its large-scale industrial system. Conversely, only seven cities received the very first NCC award in 2005, mostly eastern coastal cities, e.g., Yantai, Dalian, Qingdao, Ningbo, Xiamen, and Zhongshan, along with the western city of Baotou.

We can observe that in 2009, SO_2_ pollution in the North China Plain reduced slightly (Fig. 1b). At this time SO_2_ emissions were tightly restricted to the 5-year quantitative and binding reduction target formulated in the 11th Five-year-plan (FYP). The national SO_2_ emissions were enforced to reduce by 10% relative to the emissions by 2010 (end of 10th FYP). Most cities witnessed a reduction in SO_2_ emissions, but Chongqing faced an even larger SO_2_ pollution challenge. In the meantime, the number of civilized cities increased; newly added civilized cities included three provincial capital cities and six prefecture-level cities, mostly in the east and south.

In 2011, however, the decreasing trend of SO_2_ emissions reversed and highly polluted regions such as the North China Plain, the Yangtze River Delta and the Pearl River Delta began to worsen sharply (Figs. 1c-d). One possible explanation is that a series of preferential policies were formulated during the 12th FYP (2011–2015), causing a sharp increase in SO_2_ pollution in the first years of the 12th FYP. In fact, SO_2_ pollution dropped substantially during the latter period of the 12th FYP due to stricter targets for SO_2_ emissions reduction. Interestingly, the third batch of NCC titles was issued in early 2011, expanding civilized cities to 23 (9 provincial capitals and 14 prefecture-level cities). Later in 2011, the fourth batch was granted, increasing civilized cities by 6 provincial capitals and 22 prefecture-level cities, situated in the east, central and western China.

In 2017, SO_2_ pollution was substantially mitigated, indicating that the binding reduction targets were successfully achieved nationwide (Fig. 1e). Notably, the highly polluted North China Plain also witnessed a noteworthy reduction, which was primarily attributed to joint efforts of both sides that local governments reinforced the stringency of environmental regulation and local heavy industries upgraded their pollution control technologies. Hence, SO_2_ has not yet been a main source of environmental pollutants in China so far. Whereas by 2017, newly added civilized cities increased to 35 (5 capital cities and 30 prefecture-level). A few western cities were also granted the honorary title due to their improved environments and relatively high economic levels.

From Fig. 1 we can infer that SO_2_ emissions of 283 Chinese cities are not randomly distributed in geography. In other words, we observe that cities with high SO_2_ emissions are clustered, for example, in the North China Plain, and cities with low emissions are in the south. We next performed a Moran’s I test with k-nearest neighbors matrix (i.e., k6) [38] since it is the most appropriate matrix in the spatial Durbin DID model. Results of Moran’s I are presented in Table 2.

**Table 2:** Results of Moran’s I test

Panel A: Yearly Moran’s I from 2003 to 2018								
Year	Moran’s I	Year	Moran’s I	Year	Moran’s I	Year	Moran’s I	
2003	0.152^***^	2007	0.145^***^	2011	0.198^***^	2015	0.204^***^	
2004	0.148^***^	2008	0.153^***^	2012	0.224^***^	2016	0.251^***^	
2005	0.169^***^	2009	0.149^***^	2013	0.222^***^	2017	0.186^***^	
2006	0.154^***^	2010	0.152^***^	2014	0.230^***^	2018	0.182^***^	
Panel B: Panel Moran’s I with different matrices								
Matrix	inv300	inv350	inv400	inv450	k5	k6	k7	k8
Moran’s I	0.163^***^	0.161^***^	0.169^***^	0.164^***^	0.122^***^	0.141^***^	0.155^***^	0.160^***^

^***^p < 0.01

From Table 2, we notice that all Moran’s I values are statistically significant and positive, indicating strong spatial spillovers during the sample period, that is, the observations are not independent from each other, which violates the assumptions of classical econometrics. Besides, we also perform panel Moran’s I with different matrices and the results show that they are all significant and positive. Hence, spatial spillovers should be controlled for; we suggest that the spatial Durbin DID model will be more appropriate than the classical DID model, especially when evaluating the demonstration effect between Chinese cites. Similarly, we note that the civilized cities tend to be clustered in specific regions, such as urban agglomerations, provincial capital cities and their satellite cities. Demonstration effects is exemplified by the civilized cities which initially won the NCC award and would then stimulate neighboring cities to actively participate in the campaign and resultingly mitigate SO_2_ pollution.

### Results of classical and spatial Durbin DID models

We first estimated the classical DID models and give summarized results in Table 3. Model (1) in the second column of Table 3 only controls for city fixed effects. Model (2) in the third column consists of the two-way fixed effects classical DID model. Model (3) incorporates control variables based on Model (2). Given the low number of first and second batches of civilized cities, this may lead to biased conclusions. To conduct a robustness check, we have deleted the first batch of civilized cities from Model (4) to avoid biased estimates in the classical DID models with multiple time periods. Similarly, we deleted both the first and second batches of civilized cities from Model (5).

**Table 3:** Results of classical DID models

Variable	Model (1)	Model (2)	Model (3)	Model (4)	Model (5)
*DID*	-0.828^***^	-0.160^**^	-0.153^**^	-0.160^**^	-0.149^**^
	(0.0620)	(0.0666)	(0.0668)	(0.0704)	(0.0751)
*LnPop*			-0.121	-0.0571	-0.0784
			(0.101)	(0.0991)	(0.112)
*GDPgrate*			0.0128^**^	0.0129^**^	0.0129^**^
			(0.00504)	(0.00514)	(0.00528)
*LnFDI*			-0.00992	-0.0108	-0.0101
			(0.0123)	(0.0124)	(0.0126)
*LnGDP*			-0.0156	0.00560	0.0167
			(0.0664)	(0.0670)	(0.0690)
*CityFE*	Yes	Yes	Yes	Yes	Yes
*YearFE*	No	Yes	Yes	Yes	Yes
Obs	4528	4528	4528	4400	4256
*R*^2^	0.668	0.811	0.812	0.812	0.812

Standard errors in parentheses. ^**^ *p* < 0.05, ^***^ *p* < 0.01. CityFE and YearFE indicate city and year fixed effects, respectively

From the results of city fixed effects model (Model (1)), we observe that the estimated coefficient of the DID term (-0.828) is highly significant and negative. Model (2) also exhibits a significant and negative coefficient. However, it has a smaller reduction effect (-0.160) than that of Model (1), implying that the omission of time fixed effects leads to upward bias in Model (1). It still has significant and negative estimated coefficients in Models (3)-(5) when controlling for explanatory variables or deleting the first and second batches of civilized cities. From the above estimation results, we can conclude that the civilized cities can significantly reduce SO_2_ emissions.

The Moran’s I test results in Table 2 show strong spatial spillovers, indicating that the classical DID model may not be suitable for our study. Instead, the spatial Durbin DID models may be a better fit since they can capture spatial spillovers by incorporating spatially lagged dependent variables and spatially lagged control variables. However, since we do not yet know which model is the best fitted among spatial lag DID model, spatial error DID model, and spatial Durbin DID model, we have considered different spatial weights matrices and performed Lagrange multiplier (LM) tests to select the best one. The results of the LM tests are summarized in Table 4.

**Table 4:** Lagrange multiplier tests with different spatial weights matrices

Weights	inv300	inv350	inv400	inv450	k5	k6	k7	k8
LM test no spatial lag	524.364 [0.000]	564.675 [0.000]	605.626 [0.000]	620.997 [0.000]	1379.698 [0.000]	1663.102 [0.000]	1923.161 [0.000]	2190.663 [0.000]
robust LM test no spatial lag	3.121 [0.077]	3.915 [0.048]	4.286 [0.038]	5.163 [0.023]	40.835 [0.000]	57.428 [0.000]	68.719 [0.000]	68.232 [0.000]
LM test no spatial error	521.311 [0.000]	560.791 [0.000]	601.371 [0.000]	615.834 [0.000]	1351.681 [0.000]	1625.205 [0.000]	1877.567 [0.000]	2142.282 [0.000]
robust LM test no spatial error	0.068 [0.795]	0.030 [0.863]	0.031 [0.861]	0.001 [0.982]	12.818 [0.000]	19.532 [0.000]	23.125 [0.000]	19.850 [0.000]

*p*-values in brackets

From Table 4 we can observe that the results of the LM test statistics with inv300, inv350, inv400, and inv450 matrices support spatial lag models and reject spatial error models. However, the four LM test statistics with k5-k8 matrices are highly significant, indicating that both spatial lag model and spatial error model are appropriate; this finding is not in line with the results with inverse distance matrices.

We therefore conducted a Wald test to examine if the spatial Durbin model is better than the two above models. The Wald test results showed that the null hypotheses that: spatial Durbin model can be simplified to spatial lag model or spatial error model, can be strongly rejected at a 1% significance level, indicating that the spatial Durbin model is the best fit. Estimation results of two-way fixed effects spatial Durbin DID model with different spatial weights matrices are presented in Table 5.

**Table 5:** Results of spatial Durbin DID models

Variable	Model (6)	Model (7)	Model (8)	Model (9)	Model (10)	Model (11)	Model (12)	Model (13)
	inv300	inv350	inv400	inv450	k5	k6	k7	k8
*DID*	-0.115^***^	-0.116^***^	-0.116^***^	-0.114^***^	-0.125^***^	-0.114^***^	-0.126^***^	-0.127^***^
	(-3.492)	(-3.513)	(-3.514)	(-3.476)	(-3.789)	(-3.449)	(-3.795)	(-3.812)
*LnPop*	-0.059^*^	-0.058	-0.057	-0.054	-0.067	-0.068	-0.063	-0.063
	(-1.191)	(-1.184)	(-1.157)	(-1.101)	(-1.364)	(-1.386)	(-1.288)	(-1.263)
*GDPrate*	0.014^***^	0.014^***^	0.014^***^	0.013^***^	0.011^***^	0.012^***^	0.012^***^	0.014^***^
	(4.526)	(4.443)	(4.449)	(4.368)	(3.553)	(3.786)	(3.848)	(4.509)
*LnFDI*	-0.010	-0.010	-0.010	-0.009	-0.011	-0.013^*^	-0.012*	-0.011
	(-1.436)	(-1.398)	(-1.380)	(-1.376)	(-1.645)	(-1.844)	(-1.687)	(-1.629)
*LnGDP*	-0.068	-0.073^*^	-0.072^*^	-0.072*	-0.066	-0.060	-0.065	-0.068
	(-1.577)	(-1.682)	(-1.677)	(-1.670)	(-1.521)	(-1.384)	(-1.490)	(-1.545)
W*Lnpop	0.095	0.108	0.117	0.099	0.129	0.240^**^	0.196*	0.174
	(0.858)	(0.928)	(0.962)	(0.789)	(1.320)	(2.213)	(1.772)	(1.416)
W*GDPrate	-0.011^**^	-0.011^**^	-0.012^**^	-0.012^**^	-0.004	-0.007	-0.008	-0.014^***^
	(-2.382)	(-2.301)	(-2.416)	(-2.327)	(-0.865)	(-1.422)	(-1.466)	(-2.589)
W*LnFDI	-0.009	-0.011	-0.011	-0.012)	0.002	0.008	-0.004	0.000
	(-0.701)	(-0.843)	(-0.806)	(-0.866)	(0.155)	(0.592)	(-0.261)	(-0.001)
W*LnGDP	0.171^**^	0.190^**^	0.192**	0.201^**^	0.122	0.087	0.134	0.180^**^
	(2.357)	(2.513)	(2.467)	(2.518)	(1.649)	(1.102)	(1.597)	(2.058)
W*DID	-0.202^***^	-0.225^***^	-0.245^***^	-0.265^***^	-0.161^**^	-0.221^***^	-0.202^***^	-0.216^***^
	(-2.831)	(-2.980)	(-3.086)	(-3.201)	(-2.467)	(-3.114)	(-2.702)	(-2.752)
*ρ*	0.407^***^	0.436^***^	0.471^***^	0.482^***^	0.402^***^	0.436^***^	0.438^***^	0.445^***^
	(19.427)	(19.958)	(21.194)	(20.997)	(20.995)	(21.945)	(20.756)	(20.080)
*CityFE*	Yes	Yes	Yes	Yes	Yes	Yes	Yes	Yes
*YearFE*	Yes	Yes	Yes	Yes	Yes	Yes	Yes	Yes
Loglikelihood	-3202.139	-3193.768	-3184.807	-3181.771	-3167.200	-3163.526	-3173.439	-3184.772
Obs	4528	4528	4528	4528	4528	4528	4528	4528
*R*^2^	0.834	0.835	0.836	0.836	0.838	0.838	0.837	0.835

t statistics in parentheses. ^*^ *p* < 0.10, ^**^ *p* < 0.05, ^***^ *p* < 0.01

From Table 5, we can observe that the estimated spatial autoregressive coefficients (i.e., *ρ*) of all spatial Durbin DID models are highly significant and positive, despite slight differences in magnitude. The indication here is that increases in SO_2_ emissions of neighboring cities lead to the rise of SO_2_ emissions in own city. In other words, the highly SO_2_ polluted cities are surrounded by cities with high SO_2_ emissions, while cities with low SO_2_ emissions tend to be clustered. We also performed a log-likelihood ratio test to examine the spatial Durbin DID model against the classical DID model. The null hypothesis can be strongly rejected at the 1% significance level, indicating that the spatial Durbin DID model is a better fit.

Whereas we also notice that the estimated spatial autoregressive coefficients increase as spatial weights matrices vary with distance. Specifically, the coefficients in Models (6)-(9) range from 0.407 to 0.482 when the cut-off distance increases from 300 to 450 km. This is because the longer the distance, the more that cities are enclosed, and the stronger are the spatial spillover effects. Similar conclusions are reached for Models (10)-(13), in which the coefficients get larger as the number of neighbors increases. Since Models (6)-(13) have similar conclusions, the log-likelihood statistic can help us determine which one is the best. We have selected Model (11) as the best fit, which we will discuss later.

## Discussion

With regard to the estimated *DID* coefficient in the spatial Durbin DID model (Model 11), we find that it is still significant and negative (-0.114) and is smaller than that (-0.153) in the classical DID model because the omission of spatial spillovers can lead to biased estimates, specifically upward bias. The estimation result indicates that the civilized cities could significantly reduce SO_2_ emissions because they are highly aware of the honorary title and tend to implement strict environmental regulation to mitigate SO_2_ pollution and improve the environment for the purpose of maintaining the award in the process of review in subsequent evaluations. Otherwise, the title will be revoked if they do not meet the environmental standards. Specifically, in the campaign to maintain the golden title, the local government reinforces environmental regulations, notably for highly polluting firms, to curb emissions and strengthen public environmental facilities for pollution control. Local citizens are also encouraged to participate in environmental management to avoid environmental worsening or occurrence of environmental events. In sum, the place-based NCC policy can stimulate local governments to allocate resources effectively and implement regulation through policy intervention [39] to safeguard ‘the public good’, by reducing environmental hazards that have long been ignored [40]. It is therefore conducive to focus on SO_2_ reduction and the environment and realize a win–win situation for the promotion probability of local officials and environmental benefits of the city.

More importantly, we notice that the spatial lag of *DID*, namely, W*DID also has a significant and negative coefficient, indicating that neighboring civilized cities also contribute to reducing SO_2_ emissions of their own city. We can verify that the NCC policy can generate positive externalities, as has been confirmed by other researchers (see, amongst others, [18, 41]). One possible explanation is that neighboring cities receiving NCC award will also stimulate their own city to take stringent action to reduce SO_2_ emissions and protect the environment due to demonstration and competition effects. If a municipal government is finally granted the title, it can serve as a benchmark for neighboring municipal governments [12]. Spatial spillovers are effective in the yardstick competition and exert powerful influence towards total reductions in SO_2_ emissions. All in all, the exemplary role is beneficial to environmental quality improvements across the administrative regions.

### Robustness check

#### Replacing the dependent variable

To confirm our conclusions in a robustness check, we replace the dependent variable, SO_2_ emissions, with satellite observed SO_2_ concentrations and repeat the estimation of the spatial Durbin DID models with different spatial weights matrices. Results are reported in Table 6.

**Table 6:** Results of spatial Durbin DID models (SO_2_ concentrations)

Variable	Model (14)	Model (15)	Model (16)	Model (17)	Model (18)	Model (19)	Model (20)	Model (21)
	inv300	inv350	inv400	inv450	k5	k6	k7	k8
*DID*	-0.014^**^	-0.014^**^	-0.014^**^	-0.014^**^	-0.012^*^	-0.010	-0.010	-0.012^*^
	(-2.139)	(-2.131)	(-2.118)	(-2.113)	(-1.781)	(-1.555)	(-1.575)	(-1.773)
W*DID	-0.039^***^	-0.039^***^	-0.038^***^	-0.038^***^	-0.039^***^	-0.041***	-0.056^***^	-0.047^***^
	(-2.706)	(-2.692)	(-2.668)	(-2.659)	(-2.883)	(-2.798)	(-3.737)	(-3.017)
*ρ*	0.574^***^	0.579^***^	0.587^***^	0.590^***^	0.528^***^	0.564^***^	0.604^***^	0.613^***^
	(31.446	(31.963	(32.810)	(33.135)	(29.259)	(30.778)	(33.104)	(32.338)
*Control*	Yes	Yes	Yes	Yes	Yes	Yes	Yes	Yes
*CityFE*	Yes	Yes	Yes	Yes	Yes	Yes	Yes	Yes
*YearFE*	Yes	Yes	Yes	Yes	Yes	Yes	Yes	Yes
Log-likelihood	3726.815	3731.024	3731.228	3731.675	3681.069	3728.839	3766.689	3778.434
Obs	3836	3836	3836	3836	3836	3836	3836	3836
*R*^2^	0.856	0.856	0.856	0.857	0.852	0.856	0.859	0.859

t statistics in parentheses. ^*^p < 0.10, ^**^p < 0.05, ^***^p < 0.01. Control denotes control variables

In Table 6 the spatial autoregressive coefficients become larger than those in Table 5 because SO_2_ concentrations of Chinese cities present much stronger spatial spillovers compared with SO_2_ emissions. However, we also notice that both the *DID* and W*DID terms are still significant and negative, and consistent with those in Table 6, although the estimated spatial autoregressive coefficients (e.g., *ρ*) in the two models with two types of the dependent variable are differentiated in magnitude because they have different units. From the above analysis, it follows that the conclusions are robust and convincing.

Apart from SO_2_ pollution, to confirm the robust conclusions again we introduce satellite-observed NO_2_ concentration data as the dependent variable and then repeat the estimation of the spatial Durbin DID models. The results are presented in Table 7.

**Table 7:** Results of spatial Durbin DID models (NO_2_ concentrations)

Variable	Model (22)	Model (23)	Model (24)	Model (25)	Model (26)	Model (27)	Model (28)	Model (29)
	inv300	inv350	inv400	inv450	k5	k6	k7	k8
*DID*	-0.031^***^	-0.037^***^	-0.039^***^	-0.040^***^	-0.022^***^	-0.017^***^	-0.019^***^	-0.022^***^
	(-6.012)	(-6.902)	(-7.047)	(-7.057)	(-4.248)	(-3.344)	(-3.737)	(-4.248)
W*DID	-0.036^***^	-0.015	-0.023	-0.022	-0.055^***^	-0.047^***^	-0.047^***^	-0.055^***^
	(-2.626)	(-0.985)	(-1.352)	(-1.198)	(-4.105)	(-4.200)	(-3.767)	(-4.105)
*ρ*	0.855^***^	0.870^***^	0.887^***^	0.901^***^	0.838^***^	0.822^***^	0.829^***^	0.838^***^
	(82.114)	(80.422)	(81.111)	(82.045)	(78.707)	(81.992)	(80.000)	(78.707)
*Control*	Yes	Yes	Yes	Yes	Yes	Yes	Yes	Yes
*CityFE*	Yes	Yes	Yes	Yes	Yes	Yes	Yes	Yes
*YearFE*	Yes	Yes	Yes	Yes	Yes	Yes	Yes	Yes
Loglikelihood	4360.95	4282.01	4222.56	4171.27	4369.57	4455.73	4408.53	4369.57
*Obs*	3710	3710	3710	3710	3710	3710	3710	3710
*R*^2^	0.165	0.133	0.127	0.123	0.162	0.179	0.167	0.162

t statistics in parentheses. ^*^p < 0.10, ^***^p < 0.01

As shown in Table 7, we observe that both the *DID* and W*DID terms are still significant and negative, indicating that the NCC policy can also contribute to reducing NO_2_ pollution. In other words, the conclusions have been confirmed, again. Besides, we notice that the spatial autoregressive coefficients in Table 7 are larger than those in Table 6, implying stronger spatial spillovers of NO_2_ pollution.

#### Controlling for fiscal autonomy

On the basis of Fig. 1, economically developed cities are more likely to be granted the honorary title, since the economy is somewhat of a prerequisite for candidacy, even though economy-related indicators account for less than 3% of the total. Better economic and fiscal conditions enable these municipal governments to have more resources and stronger incentives to participate in the campaign and higher abilities to cope with environmental problems like SO_2_ pollution. To rule out the possibility of the impact of fiscal conditions on SO_2_ pollution, we follow Chen’s [42] study and introduce a fiscal autonomy variable (*Fisc*) to control for the effect, which is defined as the ratio of fiscal revenue to fiscal expenditure. In addition, the higher the fiscal autonomy, the better are fiscal conditions. If the *DID* and W*DID terms turn out to be insignificant when the *Fisc* variable and its spatial lag are incorporated in the spatial Durbin DID model, the implication is that fiscal conditions are the key contributors to SO_2_ pollution mitigation rather than the effect of the NCC policy. The estimation results are presented in Table 8.

**Table 8:** Results of spatial Durbin DID models (Alternative explanation)

Variable	Model (30)	Model (31)	Model (32)	Model (33)	Model (34)	Model (35)	Model (36)	Model (37)
	inv300	inv350	inv400	inv450	k5	k6	k7	k8
*DID*	-0.115^***^	-0.115^***^	-0.115^***^	-0.114^***^	-0.124^***^	-0.115^***^	-0.126^***^	-0.127^***^
	(-3.470)	(-3.495)	(-3.504)	(-3.464)	(-3.775)	(-3.466)	(-3.799)	(-3.810)
W*DID	-0.198^***^	-0.220^***^	-0.240^***^	-0.259^***^	-0.160^**^	-0.221^***^	-0.196^***^	-0.206^***^
	(-2.777)	(-2.906)	(-3.005)	(-3.117)	(-2.454)	(-3.105)	(-2.624)	(-2.620)
*ρ*	0.410^***^	0.433^***^	0.459^***^	0.473^***^	0.399^***^	0.424^***^	0.434^***^	0.458^***^
	(19.630)	(19.763)	(20.380)	(20.396)	(20.779)	(21.074)	(20.478)	(20.984)
*Control*	Yes	Yes	Yes	Yes	Yes	Yes	Yes	Yes
*CityFE*	Yes	Yes	Yes	Yes	Yes	Yes	Yes	Yes
*YearFE*	Yes	Yes	Yes	Yes	Yes	Yes	Yes	Yes
Loglikelihood	-3197.467	-3188.256	-3179.747	-3177.169	-3163.836	-3160.525	-3170.769	-3179.693
*Obs*	4528	4528	4528	4528	4528	4528	4528	4528
*R*^*2*^	0.835	0.835	0.836	0.836	0.838	0.838	0.837	0.836

t statistics in parentheses. ^*^p < 0.10, ^**^p < 0.05, ^***^p < 0.01

From Table 8, we observe that the variable of *Fisc* is highly significant and positive, indicating that higher fiscal autonomy means more pollution. One possible interpretation is that high fiscal autonomy not only indicates that local fiscal revenue dominates the fiscal structure of the own city, but also implies less dependence on subsidies from higher level governments. In this regard, facing the trade-off between economic growth and the environment, local governments may have fewer incentives to further mitigate SO_2_ pollution. Instead, they have more incentives to pursue better economic performance. In this way preferential policies are implemented to attract more businesses in a bid to garner more taxes, which increases the number of businesses and leads to higher SO_2_ emissions. Whereas the spatial lag of *Fisc*, namely, W*Fisc, is found to have a significant and negative impact; the implication here is that an increase in fiscal autonomy of neighboring cities leads to the decline of SO_2_ pollution of the own city. One possible reason is that the number of businesses in nearby cities decreases accordingly due to the competition effect, so that SO_2_ emissions also decline.

Most importantly, we notice that both the *DID* term and its spatial lag (W*DID) are still significant and negative after controlling for the fiscal autonomy variable, once again confirming the effective impact of the NCC policy on SO_2_ reduction. In other words, the above conclusions are robust and convincing.

#### Excluding interference from other policies

Considering that other possible environmental policies may work during the same period that interfere with our conclusions, we consider another policy implemented by the government. Specifically, 81 cities were officially granted as the new energy demonstration cities in January 2014 [43]. We refer to it as the new energy demonstration city pilot policy. Then, we control for these dummy variables (*DID_NED*) to verify if it may interact with the NCC policy to affect SO_2_ pollution. The results are presented in Table 9.

**Table 9:** Results of spatial Durbin DID models (New energy demonstration city pilot policy)

*Variable*	Model (38)	Model (31)	Model (32)	Model (33)	Model (34)	Model (35)	Model (36)	Model (37)
	inv300	inv350	inv400	inv450	k5	k6	k7	k8
DID	-0.110^***^	-0.115^***^	-0.119^***^	-0.117^***^	-0.120^***^	-0.110^***^	-0.123^***^	-0.120^***^
	(-3.452)	(-3.622)	(-3.755)	(-3.699)	(-3.740)	(-3.434)	(-3.861)	(-3.740)
DID_NED	-0.092^**^	-0.093^**^	-0.093^***^	-0.092^**^	-0.078^**^	-0.087^**^	-0.079^**^	-0.078^**^
	(-2.533)	(-2.560)	(-2.588)	(-2.536)	(-2.170)	(-2.408)	(-2.203)	(-2.170)
DID	-0.255^***^	-0.287^***^	-0.293^***^	-0.335^***^	-0.196^**^	-0.196^***^	-0.152^**^	-0.196^**^
	(-3.135)	(-3.194)	(-2.975)	(-3.167)	(-2.571)	(-2.890)	(-2.105)	(-2.571)
*ρ*	0.483^***^	0.533^***^	0.575^***^	0.608^***^	0.481^***^	0.448^***^	0.473^***^	0.481^***^
	(22.620)	(23.448)	(24.383)	(24.787)	(23.349)	(23.632)	(23.916)	(23.349)
*Control*	Yes	Yes	Yes	Yes	Yes	Yes	Yes	Yes
*CityFE*	Yes	Yes	Yes	Yes	Yes	Yes	Yes	Yes
*YearFE*	Yes	Yes	Yes	Yes	Yes	Yes	Yes	Yes
Loglikelihood	-3017.19	-3008.79	-3000.794	-2996.84	-3003.75	-2994.02	-2993.94	-3003.75
*Obs*	4528	4528	4528	4528	4528	4528	4528	4528
*R*^*2*^	0.052	0.033	0.026	0.036	0.112	0.255	0.207	0.112

t statistics in parentheses. ^**^p < 0.05, ^***^p < 0.01

We observe from Table 9 that the new variable *DID_NED* is highly significant. Moreover, the DID term remains significant and negative, indicating that our conclusions are robust.

#### Parallel trend test

**Fig. 2:**
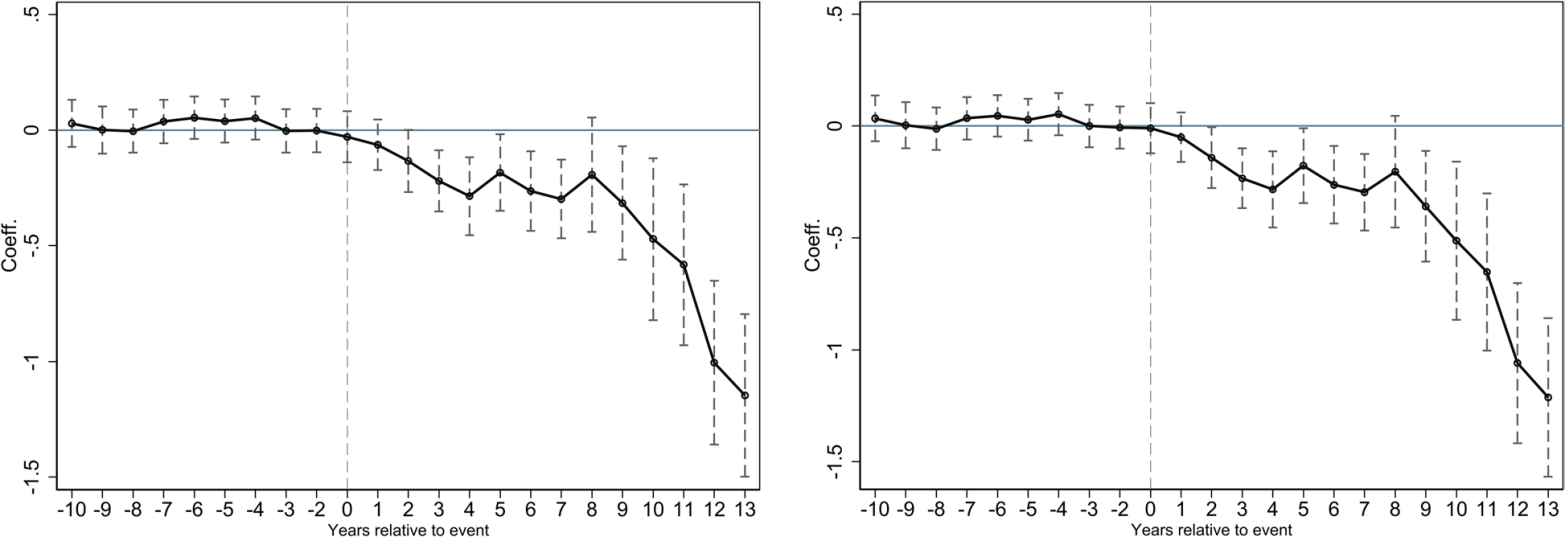
Parallel trend test results: K6 matrix vs. inv300 matrix

A prerequisite for applying the DID model is that both control and treatment groups comply with the parallel trend hypothesis. In other words, the differences of SO_2_ pollution between the two groups are fixed before the NCC policy implementation. Then, the gaps began to happen after the policy was implemented. To verify the hypothesis, we follow Beck et al. [44] by introducing a set of dummies into the spatial Durbin DID model to observe the variations between control and treatment groups. The model can be rewritten as follows.

6 $${LnSO}_{2it}={\alpha }^{\mathrm{^{\prime}}}+\sum_{{\text{k}}=-10,{\text{k}}\ne -1}^{13}{\zeta {\prime}}_{k}{DID}_{it}^{k}+\rho {\prime}W{LnSO}_{2it}+{X}_{it}\beta {\prime}+{WX}_{it}\theta {\prime}+{\mu }_{i}+{\lambda }_{t}+{\varepsilon }_{it}$$

where ${DID}_{it}^{k}$ represents a set of dummies. k is the k-th year before or after the NCC policy. If the trend of the estimated coefficients ${\zeta {\prime}}_{k}$ fluctuates around 0 when k < 0, the common trend assumption is satisfied. In contrast, if the trend is decreasing when k > 0, it indicates that a striking difference between control and treatment groups can be found after the policy. In other words, the policy began to work. The parallel trend test results are plotted in Fig. 2.

As shown in Fig. 2, the estimated coefficients ${{\zeta }{\prime}}_{k}$ fluctuated around 0 when k < 0. In other words, they are insignificant before the NCC policy was implemented. It implies that the common trend assumption is confirmed. After the policy was implemented (k > 0), the estimated coefficients are negative and significant, indicating that it works well in reducing SO_2_ pollution.

#### Placebo test

**Fig. 3:**
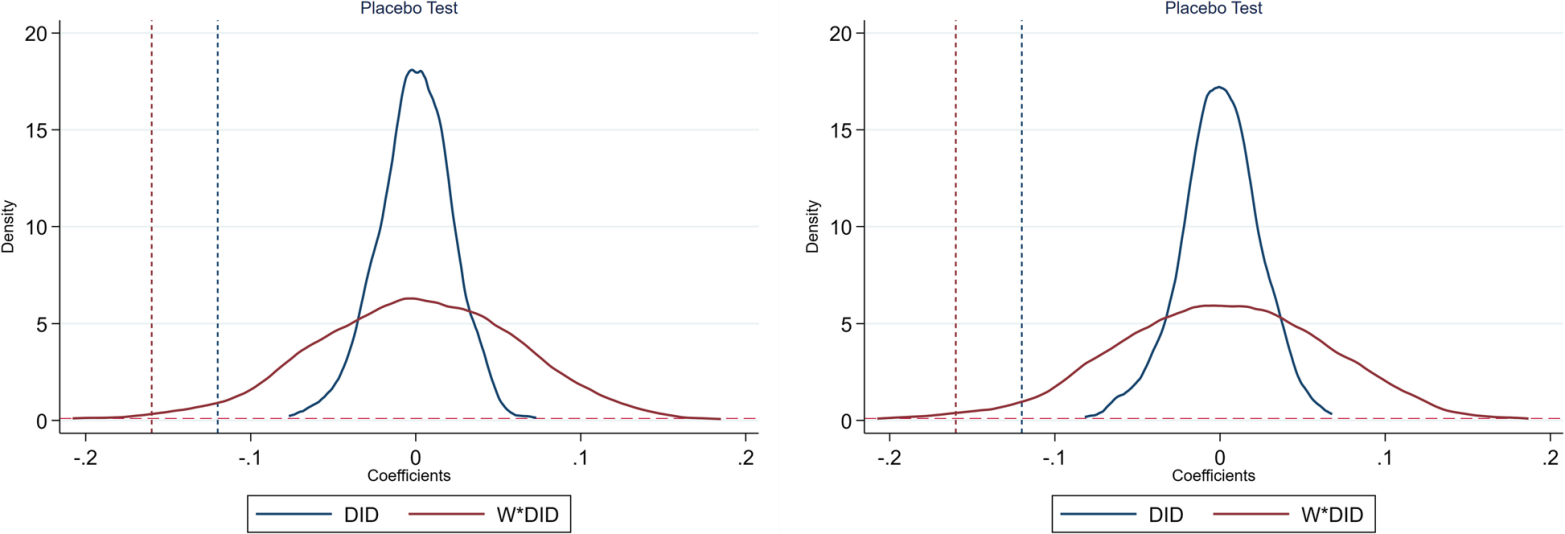
Placebo test results: K6 matrix vs. inv300 matrix

A further alternative robustness check to confirm our conclusions is to perform a placebo test. We followed the practice of Ferrara et al. [45] to randomly generate a new DID variable (*DID_P*) by randomly selecting the treatment group from the total samples while keeping the ratio of treatment observations the same as estimation results in Table 5. In the pseudo treatment group these cities did not really win the NCC title; the main aim of the placebo test is to confirm if the variable of *DID_P* is highly insignificant. In this test, random sampling and re-estimation of spatial DID models is repeated 1000 times. On the one hand, if it remains significant, the results in Table 5 could have happened by chance. On the other hand, if it is insignificant, the reduction effect of the NCC policy on SO_2_ can be confirmed. We applied the kernel density estimation method to plot the densities of the coefficients of the *DID* and W*DID variables for 1000 times, as shown in Fig. 3.

The blue and red dash lines are the estimated coefficients of the *DID* and W*DID variables, respectively. We find that the DID coefficient of sampling regression is close to 0. Furthermore, the *p* values are all greater than 10%, indicating that the pseudo civilized cities cannot significantly reduce local SO_2_ pollution. Similarly, we notice that the W*DID coefficient is also close to 0. However, the far-left side of the density curve of W*DID is approaching the estimated coefficient. The main reason for this is that low probability events have happened, but they do not undermine the main conclusions. In this sense, we can conclude that civilized cities can also contribute to mitigating SO_2_ pollution of neighboring cities.

To better understand the random selection of the placebo test, the results of one random sampling regression are presented in Table 10.

**Table 10:** Placebo test results (Random DID)

Variable	Model (38)	Model (39)	Model (40)	Model (41)	Model (42)	Model (43)	Model (44)	Model (45)
	inv300	inv350	inv400	inv450	k5	k6	k7	k8
*DID_P*	0.001	0.000	0.000	0.000	-0.003	-0.005	-0.005	-0.006
	(0.037)	(0.006)	(-0.008)	(-0.003)	(-0.135)	(-0.242	(-0.201)	(-0.264)
W*DID_P	0.011	0.013	0.026	0.016	0.036	0.059	0.088	0.055
	(0.186)	(0.216)	(0.397)	(0.228)	(0.735)	(1.100)	(1.511)	(0.877)
*ρ*	0.411^***^	0.450^***^	0.461^***^	0.484^***^	0.402^***^	0.431^***^	0.442^***^	0.443^***^
	(19.669)	(20.873)	(20.465)	(21.081)	(20.977)	(21.550)	(21.013)	(19.913)
*Control*	Yes	Yes	Yes	Yes	Yes	Yes	Yes	Yes
*CityFE*	Yes	Yes	Yes	Yes	Yes	Yes	Yes	Yes
*YearFE*	Yes	Yes	Yes	Yes	Yes	Yes	Yes	Yes
Loglikelihood	-3214.488	-3205.640	-3198.102	-3194.616	-3179.736	-3176.374	-3185.984	-3198.828
Obs	4528	4528	4528	4528	4528	4528	4528	4528
*R*^2^	0.834	0.834	0.835	0.835	0.837	0.837	0.836	0.834

t statistics in parentheses. ^***^p < 0.01

We notice that the estimated coefficients of the *DID_P* variable in Model (30)-(37) are all highly insignificant. In addition, the spatial lag (W*DID_P) is also found to be statistically insignificant. Thus, we can rule out the possibility of a random event: the findings of the present research are robust and convincing.

#### Mechanism analysis

The above conclusions indicate that the NCC policy can contribute to reducing pollution. Then, a question may arise through which channels does it mitigate pollution? From the above analysis, civilized cities reduce pollution by lowering the share of the secondary industry since it is the largest pollutant emitter in China [46]. In other words, industrial structure upgrading is the key channel for civilized cities to reduce pollution. Hence, we focus on testing for the influence path of industrial structure upgrading on SO_2_ pollution. Specifically, it is measured by the share of the secondary industry to GDP (*Second*). The estimation results are presented in Table 11.

**Table 11:** Mechanism analysis: Industrial structure upgrading

Variable	Inv300	Inv350	K5	K6
	Model (46)	Model (47)	Model (48)	Model (49
DID	-0.074^**^	-0.069^**^	-0.071^**^	-0.073^**^
	(-2.205)	(-2.045)	(-2.107)	(-2.173)
Second	0.011^***^	0.013^***^	0.011^***^	0.012^***^
	(5.297)	(6.056)	(5.201)	(5.297)
W*DID	-0.372^***^	-0.412^***^	-0.349^***^	-0.336^***^
	(-2.205)	(-4.542)	(-4.608)	(-3.837)
W* Second	-0.020^***^	-0.023^***^	-0.019^***^	-0.018^***^
	(-4.592)	(-5.609)	(-4.844)	(-4.245)
*ρ*	0.520^***^	0.472^***^	0.432^***^	0.466^***^
	(19.947)	(19.297)	(20.098)	(19.736)
*Control*	Yes	Yes	Yes	Yes
YearFE	Yes	Yes	Yes	Yes
CityFE	Yes	Yes	Yes	Yes
LR	-2295.11	-2296.45	-2281.42	-2296.76
Obs	3710	3710	3710	3710
*R*^2^	0.003	0.013	0.006	0.064

t statistics in parentheses. ^*^p < 0.10, ^**^p < 0.05, ^***^p < 0.01

From Model (46), we find that the variable *Second* is significant and positive, indicating that a decline in the share of the secondary industry can help reduce SO_2_ pollution. On the other hand, we notice that its spatial lag W*Second in Model (47) is significantly negative, suggesting that a decrease in the share in neighboring cities could exacerbate SO_2_ pollution. One possible interpretation is that industries cities subjected to strict environmental regulation move to neighboring cities, leading to increasing both the share of the industry and SO_2_ pollution of neighbors. Regarding the *DID* term, we find that in both Models (46) and (47) it is still significant and negative, implying that the NCC policy works. Hence, the influence channel is confirmed.

## Conclusions and policy implications

In this research we adopted the spatial Durbin DID model and panel data of 283 Chinese cities from 2003 to 2018 to analyze the local (direct) and spillover effects (indirect) of the National Civilized City (NCC) policy on SO_2_ pollution. We found that overall SO_2_ pollution presented a decreasing trend from 2003 to 2018, but with two breaks in 2007 and 2011, when the declines reversed. Meanwhile, we found that the number of civilized cities continued to increase in five batches from 2005 to 2017, with most located in the economically developed eastern region. Moreover, among 31 capital cities, 25 had won the honorary title. The results of the spatial Durbin DID model revealed that civilized cities could significantly reduce local SO_2_ emissions. In addition, neighboring civilized cities were also conducive to mitigating SO_2_ pollution of the own city, indicating that the spatial spillover effect of the NCC policy had worked. Robustness checks, including replacing the dependent variable with satellite observed SO_2_ concentrations, controlling for fiscal autonomy and the placebo test, confirmed robust and convincing conclusions.

Based on the findings of this study, policy implications can be proposed as follows. Since the NCC policy exhibits strong environmental effects, the criteria for assessing environmental quality of civilized cities should be reinforced or further improved. In addition, central government should strictly implement the selection criteria for the title and rule out those under-qualified cities with major environmental problems by revoking their titles. Given that municipal governments are incentivized to take measures to protect the environment, controlling the number of civilized cities can significantly intensify competition for the title and instigate environmental improvements. Central government should make full use of the NCC policy to push local governments to focus on reducing pollution, improving the environment, and finally making the cities better. Apart from the NCC policy, the policymakers should also develop more appropriate policies that work for different types of cities to push them to reduce pollution. For example, appropriate incentive mechanisms should be designed and implemented for those non-civilized cities to encourage them to mitigate pollutants and improve the local environment, although they are not granted the honorary title.

It should be highlighted that the spatial spillover demonstration effect of civilized cities works. Not only do civilized cities reduce local pollution, but they also exhibit a demonstration effect. After a city has won the title, neighboring cities subsequently tend to adopt similar strategies and measures for environmental protection in order to win the performance contest and be granted the golden title. However, we observed that most of the civilized cities are located in the economically developed eastern regions, which is closely related to economic levels. A better way to promote the widest range of environmental improvements in western cities is to relatively relax the selection criteria and grant the golden title to cities with high environmental standards; in so doing the demonstration effect and spillovers may be maximized to stimulate neighboring cities to also improve the environment.

Apart from measures and regulation by local governments, another way to build civilized cities with better environmental quality is to widen channels to encourage citizens to participate in environmental management. Building high quality civilized cities and solving environmental problems is a long-term campaign which cannot merely be heavily dependent on “command and control” environmental measures. We noticed that some civilized cities also suffer from environmental pollution. In response to the challenges, local governments should emphasize the importance of public participation in environmental management because it helps promote the effective implementation of environmental regulation [47]. In addition, monitoring and reporting environmental violations by the public should be encouraged. It has been evidenced that it is an effective way to address environmental problems because responding to environmental complaints from local governments has become a priority in most Chinese cities, including the NCC ones. Joint cooperation between local government and the public not only helps build environmentally friendly civilized cities, but also contributes to achieving the sustainable development goals of Chinese cities. 

## Data Availability

The datasets generated and/or analyses during the current study are available from the corresponding author on reasonable request.
